# Maternal willingness to pay for infant and young child nutrition counseling services in Vietnam

**DOI:** 10.3402/gha.v8.28001

**Published:** 2015-08-31

**Authors:** Phuong H. Nguyen, Minh V. Hoang, Nemat Hajeebhoy, Lan M. Tran, Chung H. Le, Purnima Menon, Rahul Rawat

**Affiliations:** 1 International Food Policy Research Institute, Washington, DC, USA; 2 Center for Health System Research, Hanoi Medical University, Hanoi, Vietnam; 3 FHI360, Hanoi, Vietnam

**Keywords:** willingness to pay, nutrition counseling services, infant and young child feeding practice, Vietnam

## Abstract

**Background:**

Alive & Thrive Vietnam, a 6-year initiative (2009–2014), has developed and incorporated elements of social franchising into government health services to provide high-quality nutrition counseling services to improve infant and young child feeding practices. One element of franchising that has not yet been implemented is fee for service, which is a potential financing mechanism for sustaining services in the long run.

**Objective:**

This research aims to estimate maternal willingness to pay (WTP) for nutrition counseling services and to examine potential factors associated with their WTP.

**Design and methods:**

Data were drawn from an impact evaluation survey of 2,511 women with a child <2 years old from four provinces in Vietnam. An iterative bidding technique was employed to explore individual WTP. The first bid was defined as VND 20,000 (~US$ 1), which was approximately the level of the actual service cost. Depending on the participant response, the bid increased or decreased. Finally, the respondents were asked about the highest price they would be willing to pay for the service.

**Results:**

Overall, 92.6% of clients reported a need for nutrition counseling services for children <2 years. The WTP rates at bid levels of VND 5,000, 10,000, 20,000, 40,000, and 100,000 were 95.2, 94.4, 90.7, 68.9, and 33.4%, respectively. The mean and median of the maximum WTP were VND 58,500 and 50,000, respectively. In multiple regression models, WTP rates were higher among younger women, the Kinh majority group, and better educated and wealthier women.

**Conclusion:**

A high demand for nutrition counseling coupled with a WTP by almost all segments of society would potentially cover costs of delivery for nutrition counseling services in Vietnam.

The benefits of breastfeeding to infant growth and development (1), as well as long-term health outcomes (2), are well recognized. Despite its known benefits, breastfeeding practice remains sub-optimal in developing countries (3), including Vietnam (4). Breastfeeding promotion and complementary feeding education have been highlighted in the recent Lancet series on maternal and child undernutrition as a cost-effective approach to reduce the burden of child undernutrition (5).

Alive & Thrive (A&T) is an initiative funded by the Bill & Melinda Gates Foundation aiming to reduce undernutrition and death caused by sub-optimal infant and young child feeding (IYCF) practices in Bangladesh, Ethiopia, and Vietnam. In Vietnam, A&T in partnership with the National Institute of Nutrition (NIN) has incorporated elements of franchising and established the “Mat Troi Be Tho” (MTBT) model (or Little Sun in English) to shape and support the demand for IYCF services. The MTBT franchise model has two main purposes: 1) to standardize and monitor services to ensure that counseling on IYCF is uniform and of good quality; and 2) to build on the existing healthcare infrastructure and decentralized services promoted by the Government of Vietnam to ensure reach, utilization, and sustainability. The model also focuses on good quality and relevant training of health workers to build the capacity of those who are in positions to encourage and support mothers to practice optimal IYCF (6). Findings from a process evaluation of the A&T program showed that the nutrition counseling service based on the IYCF franchise model has significantly improved the mother's breastfeeding-related practices and that mothers report satisfaction with the service they receive at the franchise (7).

Collecting fees for services is a critical element of a franchise model for three main reasons. First, worldwide experience shows that consumers tend to value services more when they pay for them; second, having a mechanism for generating revenues is an important part of attracting franchisees and incentivizing high performance; and finally, long-term sustainability of the franchise will depend on its ability to generate revenue (e.g. through the eventual establishment of franchise dues that are based on a percentage of revenues generated through client fees) that enable it to recover operating costs and pay for franchise advertising and promotions. In Vietnam, the MTBT franchises are nested in public sector health facilities. Under current laws, the Government of Vietnam levies no fees for health services (examining and treating diseases) provided to children <6 years of age through the public sector. Hence, MTBT franchises are reluctant to apply fees for service for franchise nutrition counseling service because they are not accustomed to these charging fees for such services, particularly for children. To date, nutrition counseling services have been provided for free. In the long run, however, the sustainability of the model would rely on establishment of a suitable financing mechanism such as user fees or inclusion of health insurance.

User fees would be heavily dependent on willingness to pay (WTP) of clients for the service. The WTP concept generally refers to the economic value of a good to a person or a household under given conditions (8–12). Information on WTP can be useful for designers and planners in assessing economic viability of projects, setting affordable fee, evaluating policy alternatives, assessing financial sustainability, and designing socially equitable subsidies. This article aims to estimate maternal WTP for nutrition counseling services in Vietnam and to examine the factors associated with it.

## Methods

### Data sources

Data for this study were collected within the context of a larger evaluation of A&T's interventions in Vietnam (13). The end line round was carried out between June and August 2014 in 40 communes from four provinces (Thai Nguyen, Thanh Hoa, Quang Ngai, and Vinh Long), spanning the northern, central, and southern regions of Vietnam and are geographically representative of the 15 provinces in which A&T operates.

### Sample size, sampling, and study subjects

The sample size was estimated based on the expected change in rates of stunting and different IYCF indicators as a result of the A&T intervention, 80% power to detect those differences, and an alpha level of significance of 0.05. Cluster sampling (based on communes) was applied to select a total sample size of 5,800 children aged 0–59.9 months (14). The study subjects for this study were a subset of 2,511 women who had at least one child aged 0–24 months.

### Data collection

The contingent valuation method (CVM) was applied for data collection in our study. CVM is a survey-based economic practice that asks individuals how much they are willing to pay for a particular good or service (9, 15, 16). In this approach, interviewees were given different bids before being asked about their maximum WTP for nutrition counseling services.

**Fig. 1:**
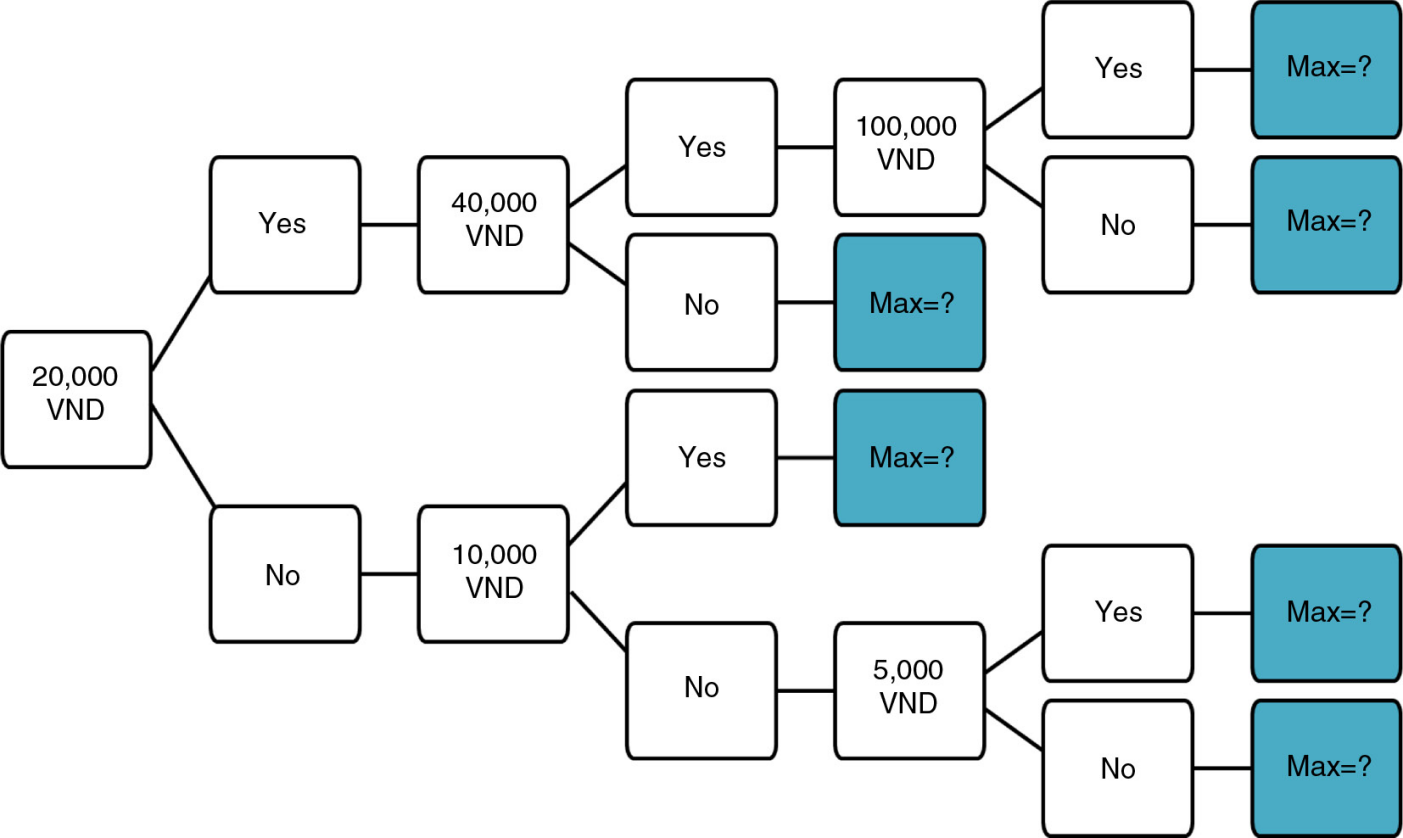
Iterative bidding technique used to elicit willingness to pay for nutrition counseling services in Vietnam. First bidding questions: If a nutrition counseling session costs VND 20,000, are you willing to pay that amount? Exchange rate: US$ 1=VND 20,000.

An iterative bidding technique was used to explore individual WTP that involved a scenario to describe the IYCF counseling service (Box 1) followed by a sequence of dichotomous choice questions (i.e. yes or no to the bid offered in each question) and a final open-ended question about the mothers’ maximum WTP (Fig. 1). Mothers were offered the service at a starting price of VND 20,000 (about US$ 1.00), which was approximately the actual cost of the service (17). For a mother who accepted the price, the bid was raised to 40,000. If she again accepted this amount, the bid was raised to 100,000. At this level, the mother was asked about her maximum WTP either if she accepted or rejected the 100,000 bid. In a corresponding way, if the mother rejected the starting bid of 20,000, the bid was lowered to 10,000. A mother who accepted this bid was asked about her maximum WTP. But for a mother, who also rejected 10,000, the bid was lowered to 5,000 and then the mother was asked about her maximum WTP. Details of bidding questions are presented in Box 2.

***Box 1*. The scenario to describe the IYCF counseling service**

Good morning/afternoon. I am ________ from the Hanoi Medical School.

We are inviting you to participate in the nutrition counseling session aiming to improve infant and young child practices (IYCF) for mothers with children <24 months old. This IYCF counseling session will take place in the ‘Mat Troi Be Tho’ counseling room in our commune health center (CHC). In each session, the trained CHC doctors or nurse will introduce one specific topic related to breastfeeding and complementary feeding practices. They will also listen to any questions or concern you may have, then advice you the best way to feed your child or support you to try the optimal practice. The CHC doctor/nurse will use the counseling card books and other materials to help you understand and remember the information. You will also be provided with a mother–baby book and leaflets where you can find many useful information for feeding your child and follow the growth of your child. The IYCF counseling session will be offered for pregnant women in the third trimester and mothers with children less than 24 months. You may already participated in this IYCF counseling session, which have been provided for free so far because we got funded from the Alive & Thrive project. However, in the future, this service will no longer get funded and we will need to collect a fee from participants. We would like to ask if you are interested in this IYCF counseling session, and if yes, how much you are willing to pay for the service based on our questions below.

***Box 2*. Willingness to pay questions**

**Table:** 

1.	Are you interested in IYCF counseling?	Yes ……………………………1 No………………………………0
2.	If one IYCF counseling session at CHC is **VND 20,000**, are you willing to pay for it?	Yes ……………………………1 > >3 No………………………………0 >> 5 Don't know, no answer…………88 > > 5
3.	If one IYCF counseling session at CHC is **VND 40,000**, are you willing to pay for it?	Yes ……………………………1 > >4 No………………………………0 >> 7 Don't know, no answer…………88 > > 7
4.	If one IYCF counseling session at CHC is **VND 100,000**, are you willing to pay for it?	Yes ……………………………1 >> 7 Don't know, no answer…………88 > > 7
5.	If one IYCF counseling session at CHC is **VND 10,000**, are you willing to pay for it?	Yes ……………………………1 > > 7 No………………………………0 >> 6 Don't know, no answer…………88 > > 6
6.	If one IYCF counseling session at CHC is **VND 5,000**, are you willing to pay for it?	Yes ……………………………1 > > 7 No………………………………0 > > 7 Don't know, no answer…………88 > >7
7.	What is the maximum VND you are willing to pay for one IYCF counseling session?	……………….VND
8.	Why are you willing to pay for IYCF counseling?	Because my child does not grow as expected (underweight, stunting………………………1 Because I do not have enough knowledge and skills to feed my child……………………….2 Because I am satisfied with the counseling service at CHC………………………….…3 Other (specifies)……………………………88
9.	Why are you NOT willing to pay for IYCF counseling?	I have relatives who are health providers that can counsel me…………….……………….1 I don't think it is necessary …….………….2 I don't have money ………………….…….3 I don't have time… …………………….….4 Others (specifies)………………………….88

In addition, we collected a number of variables that capture different domains of household socioeconomic characteristics such as the ownership of house and land, housing quality (e.g. house construction materials), and access to services (water, electricity, gas, and sanitation services). Household assets were measured by asking a number of questions on the possession of different types of durable goods, productive assets, small animals, and livestock. Wealth index quintile was derived by Principal Component Analysis (PCA) with 17 main types of durable assets and 7 housing conditions. PCA is the ‘reduction dimensions’ statistic technique that allows us to investigate the linear combination of assets in the household through variance maximization, in order to reflect the wealth index of households (18). Other information collected included mother's characteristics (age, education, and ethnicity).

### Data management and analysis

Data were entered into EpiData management software. Statistical analyses were carried out using Stata 12. Descriptive analysis was applied to report background characteristics of study participants. A logistic regression model was performed to estimate the probability of being willing to pay for the nutrition service at the bid of VND 20,000 based on socioeconomic status of the mothers. Log-linear regression model was applied to identify socioeconomic correlates that would influence the maternal WTP amount (as the data on WTP amount are not normally distributed). A significant level of 0.05 was used.

### Ethical considerations

Ethical approval was obtained from the Institutional Review Board of the Institute of the Social and Medical Studies in Hanoi and from the International Food Policy Research Institute, Washington, DC. Informed consent forms were obtained from all subjects before participating in the study.

## Results

Demographic and socioeconomic characteristics of the respondents are summarized in Table 1. Average age was 27.7 (±5.14), with the majority (58.7%) belonging to 25–34 year age group. Almost all of the women identified themselves as the Kinh (majority group) and 13.7% belong to ethnic minority groups. More than 90% of mothers had completed secondary school or higher, and their main occupation was farming (~40%).

**Table 1:** General characteristics of the study subjects

	Frequency *(n)*	%
Age		
18–24	735	29.3
25–34	1,473	58.7
35 +	303	12.1
Ethnicity		
Ethnic minority	345	13.7
Kinh	2,166	86.3
Education		
Primary	205	8.2
Secondary	1,068	42.5
Tertiary	721	28.7
Higher	517	20.6
Occupation		
Farmer	985	39.2
Salary government employee	298	11.9
Salary non-government employee	398	15.9
Small trader/self-employment	461	18.4
Housewife	353	14.1
Jobless	16	0.6
Economic status (quintile)		
First group (poorest)	475	18.9
Second group	483	19.2
Third group	499	19.9
Fourth group	535	21.3
Fifth group (richest)	519	20.7
Child age		
< 6 months	997	39.7
6–11 months	353	14.1
12–24 months	1,161	46.2
Total	2,511	100

**Fig. 2:**
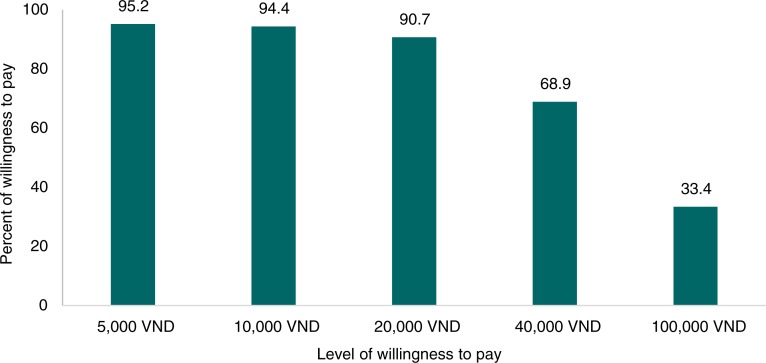
WTP rates for counseling services at different price levels. Exchange rate: US$ 1 = VND 20,000.

Overall, 92.6% of clients reported a need for nutrition counseling services, indicative of potential demand (results not shown). The rates of WTP for counseling services at different price levels are presented in Fig. 2. More than 90% of mothers report WTP for these services at a price of VND 20,000 or less, two thirds are willing to pay VND 40,000, and only a third are willing to pay 100,000 VND. Overall, the mean and median of the maximum WTP were VND 58,400 and 50,000, respectively (7.4% of women who did not need the nutrition counseling had WTP of zero).

Bivariate analyses showed that the WTP rates for counseling services at price levels of VND 20,000 were significantly associated with mother's age, education, occupation, and wealth index (Table 2). These results were confirmed in a multiple logistic regression model except for mother's occupation. Younger mothers (18–24 years) are more likely to be willing to pay for nutrition counseling services (OR: 2.26; *p*=0.001), as are mothers belonging to the Kinh ethnic group compared to other minority ethnic groups (OR: 1.5; *p*=0.04), and mothers with greater than a primary education (OR ~1.7, *p*<0.05). Additionally, those in the higher socioeconomic quintile are also more likely to be willing to pay for the nutrition counseling services. Similar results were found for other bid levels (results now shown).

**Table 2:** Logistic regression analyses of correlates of willingness to pay rate (at the bid of VND 20,000^a^)

	Bivariate associations		Multiple logistic regression	
		
	OR	95% CI	OR	95% CI
Age				
18–24^b^	2.56***	1.57; 4.16	2.26**	1.39; 3.66
25–34	1.41+	0.95; 2.09	1.26	0.85; 1.86
≥35+^b^				
Ethnicity				
Ethnic minority^b^				
Kinh	1.24	0.83; 1.86	1.48*	1.02; 2.16
Education				
Primary^b^				
Secondary	2.37***	1.56; 3.59	1.7*	1.12; 2.57
Tertiary	3.13***	1.97; 4.96	1.76*	1.08; 2.85
Higher	5.45***	3.07; 9.65	1.73	0.93; 3.23
Occupation				
Farmer^b^				
Salary government employee	3.01**	1.59; 5.68	1.29	0.68; 2.47
Salary non-government employee	2.01**	1.25; 3.23	1.32	0.81; 2.16
Small trader/self-employment	2.13**	1.34; 3.36	1.32	0.82; 2.11
Housewife	1.06	0.71; 1.59	0.76	0.49; 1.16
Jobless	0.51	0.14; 1.83	0.29	0.08; 1.09
Economic status (quintile)				
First group (poorest)^b^				
Second group	1.83**	1.23; 2.72	1.59*	1.07; 2.37
Third group	2.44***	1.59; 3.72	1.93**	1.25; 2.99
Fourth group	4.26***	2.60; 6.99	2.68***	1.62; 4.42
Fifth group (richest)	3.90***	2.40; 6.34	2.07**	1.22; 3.51
Child age				
<6 months^b^				
6–11 months	0.89	0.56; 1.40	1.01	0.64; 1.57
12–24 months	0.81	0.59; 1.12	0.93	0.68; 1.25
Constant			2.88**	1.54; 5.35

OR: odd ratio; CI: confidence interval.

+ *p*<0.10

* *p*<0.05

** *p*<0.01

*** *p*<0.001.

a Exchange rate: US$ 1=VND 20,000;

b reference group.

Results from a multiple log-linear regression model analyzing socioeconomic correlates of the maximum WTP amount revealed three significant determinant factors: younger mothers and mothers belonging to higher wealth index quintiles were willing to pay a higher amount as compared to those in the lowest quintile (Table 3). In addition, mothers working as a salaried government employee and small traders/self-employed are willing to pay a higher amount compared to farmers. Child age is neither associated with WTP rate nor WTP amount.

**Table 3:** Log-linear regression model analyzing socioeconomic correlates of the maximum willingness to pay amount

	Bivariate associations		Multiple logistic regression	
		
	Coefficient	95% CI	Coefficient	95% CI
Age				
18–24	0.29***	0.18; 0.39	0.28***	0.17; −0.39
25–34	0.13*	0.03; 0.23	0.09	−0.01; −0.19
35 + a				
Ethnicity				
Ethnic minority^a^				
Kinh	0.09+	0.00; 0.18	0.02	−0.08; 0.11
Education				
Primary^a^				
Secondary	0.22***	0.10; 0.34	0.1	−0.03; 0.22
Tertiary	0.27***	0.14; 0.39	0.06	−0.08; 0.19
Higher	0.43***	0.30; 0.56	0.12	−0.03; 0.27
Occupation				
Farmer^a^				
Salary government employee	0.35***	0.24; 0.45	0.17**	0.05; 0.30
Salary non-government employee	0.22***	0.13; 0.31	0.08	−0.02; 0.18
Small trader/self-employment	0.26***	0.17; 0.34	0.13**	0.04; 0.23
Housewife	0.06	−0.03; 0.16	−0.06	−0.16; 0.04
Jobless	0.21	−0.19; 0.61	−0.03	−0.42; 0.37
Economic status (quintile)				
First group (poorest)^a^				
Second group	0.22***	0.12; 0.32	0.18***	0.08; 0.29
Third group	0.26***	0.17; 0.36	0.21***	0.11; 0.32
Fourth group	0.37***	0.27; 0.46	0.28***	0.17; 0.39
Fifth group (richest)	0.46***	0.36; 0.56	0.35***	0.23; 0.47
Child age				
< 6 months^a^				
6–11 months	−0.08	−0.17; 0.02	−0.08	−0.17; 0.02
12–24 months	−0.07+	−0.13; 0.00	−0.03	−0.1; 0.03
Constant			3.69***	3.53; 3.84

CI: Confidence Interval.

+ p<0.10

* *p*<0.05

** *p*<0.01

*** *p*<0.001.

a Reference group.

## Discussion

Little is known about WTP for preventive nutrition counseling services in Vietnam, or elsewhere. Several studies report WTP of specific nutrition products (e.g. lipid-based nutrient supplements) or nutrition counseling services for patients (e.g. obesity, chronic diseases) but not for receipt of preventive nutrition service for infants and young children (19–21). The results from this study provide evidence for instituting user fees as potential financing mechanisms for ensuring sustainability of the model. These results also indicate that nutrition counseling services are highly valued by clients as evidenced from the high reported need and WTP. Consequently, the results call for incorporating preventive nutrition services for children within the health system in Vietnam.

We found that 90.7% of mothers in the study areas were interested in and willing to pay for preventive nutrition counseling services at the price of VND 20,000 (~US$ 1), which was about the level of the actual service cost (22). This high WTP rate reflects the fact that the nutrition counseling service was greatly appreciated by the women. Results from the process evaluation of the A&T program reported changes in quality of care at the franchises and also showed high levels of client satisfaction with the counseling skills of health providers, usefulness of the advice received, facility appearance, and counseling materials (23).

The mean of maximum WTP amount was approximately VND 58,400 (about US$ 2.7), which is approximately the cost of other routine medical checkups and examinations at commune health centers (24). In addition, the median of maximum WTP of VND 50,000 implies the reasonably high consensus among children's caregivers on the financial value of the nutrition counseling service.

Our findings also suggest that higher education or better economic status were significantly associated with higher rate of WTP for the nutrition service. Economic status was also proven to be an independently significant correlate of the WTP amount. These findings are not novel, and are expected, but show a high degree of validity of our WTP data. The findings are in line with our hypothesis that the more people understand the importance of the nutrition counseling, the more likely they are willing to pay for the service. Further, poorer households (with lower income) should have lower demand for all goods in general, including nutrition service due to income constraint. Our findings are in line with a study in Korea, showing the younger, richer, and more-educated participants were significantly more likely to be interested in nutrition topics and willing to participate in nutrition program (20). The lower WTP among worse-off households could result in a lower chance of having access to improved preventive nutrition counseling services by this vulnerable group. This suggests that the Government of Vietnam should provide special support to people with low education and/or who belong to a worse-off group, in order to facilitate access to this important service.

We hypothesized that demands for infant and young child nutrition counseling services would be varied by child age, thus affecting maternal WTP. For example, mothers of young children in the transition period from breastfeeding to complementary feeding may have greater demands to know how to introduce appropriate complementary food while still maintaining breastfeeding, thus having higher WTP. However, our findings are somewhat unexpected because we did not find the association between child age and WTP.

Although user fees are not currently being implemented in many MTBT communes, there is a great interest in exploring this mechanism to ensure the sustainability of the model. In Vietnam, there are three provisos attached to the user fee mechanism including the removal of legal restrictions on charging for health services through the public sector; WTP by women and other caregivers for counseling services; and, finally, consideration of the costs and benefits of introducing a user fee more generally. Evidence from this study addresses the core question on WTP by mothers and caregivers. Additionally, data from the study can be used to inform further economic analysis (e.g. cost-benefit, cost-effectiveness, and cost-utility) and policy dialogue on charging user fees for preventive nutrition services for children under six in Vietnam.

We need to note some methodological constraint associated with the use of the CVMs in this study. Eliciting consumer preferences through in-person interviews was not an easy task. Several potential biases might be introduced because of the way the questions were framed, the contingent valuation scenarios, the elicitation method used, and the survey method that was conducted. To overcome these challenges, we conducted several field visits in order to develop appropriate contingent valuation scenarios and questions. We also implemented a number of cognitive interviews to make sure that the contingent valuation scenarios and questions were easy to understand among the local people. Careful training of enumerators and field-testing also helped to ensure validity and reliability of the study findings.

We used the bidding game technique because it has been the most widely used in developing countries (25–27). The bidding game technique is more market realistic than a single open-ended question (28) and more reliable than a single dichotomous choice question (11). A disadvantage of the bidding game is the threat of starting-point bias, where the respondent's final WTP value is not independent of the first bid prompted by the interviewer (25, 29). The starting-point bias is known as ‘an anchoring effect’ (30), which occurs when the first bid influences the WTP amount as the respondent may consider it as a ‘normal’ value. We set up the starting point based on the actual cost of the service (22) as well on the experience from our pilot study. Our pilot showed that the starting bids of VND 5,000, VND 10,000, and VND 20,000 were accepted by 94, 92, and 91% of the respondents.

Our findings were also possibly affected by other biases such as 1) information bias, which occurs when the WTP depends on who does the interview, what information about the counseling service is provided, and what other information the respondents have about the service. We selected interviewers with good research experiences and did careful training based on the standard and clear scenario about the counseling service to minimize these biases; 2) strategic bias, which occurs when a respondent purposely states a WTP higher or lower than the true level. In this study, one respondent may think that it is better with a low estimated WTP in order to have the services provided for free also in the future. There may even be respondents stating a zero WTP as a protest against the idea of having user fees pay for the services. Another respondent may think that a high estimated WTP is good for securing the future of the services and therefore overestimate her true WTP, etc. We consider the risk of a strategic bias where women would overstate their true WTP to be unlikely because the scenario clearly explained to the mothers that fees may be used to fund these services in the future. So the mothers were aware of the fact that, in the future, they might have to pay these amounts. A strategic bias where mothers would underestimate their true WTP would, to the extent that it exists, mean an underestimation of the elicited WTP in this study. Since the elicited WTP is high relative to the cost of provision, the risk of this bias does not present a large problem for this study.

## Conclusions

There is a high demand for nutrition counseling services and a WTP by almost all segments of society for these services, which would potentially cover cost of delivery. Findings from our study provide not only evidence for establishing user fees as a potential financing mechanism, but also demonstrate that preventive nutrition counseling services are highly valued. We recommend that decision makers in Vietnam consider incorporating preventive nutrition counseling services into routine health systems.

## References

[1] Gartner LM, Morton J, Lawrence RA, Naylor AJ, O'Hare D, Schanler RJ (2005). Breastfeeding and the use of human milk. Pediatrics.

[2] Horta B, Bahl R, Martines J, Victora C (2007). Evidence on the long-term effects of breastfeeding: systematic reviews and meta-analysis.

[3] Lauer JA, Betran AP, Victora CG, de Onis M, Barros AJ (2004). Breastfeeding patterns and exposure to suboptimal breastfeeding among children in developing countries: review and analysis of nationally representative surveys. BMC Med.

[4] Dibley MJ, Senarath U, Agho KE (2010). Infant and young child feeding indicators across nine East and Southeast Asian countries: an analysis of National Survey Data 2000–2005. Public Health Nutr.

[5] Bhutta ZA, Ahmed T, Black RE, Cousens S, Dewey K, Giugliani E (2008). What works? Interventions for maternal and child undernutrition and survival. Lancet.

[6] Baker J, Sanghvi T, Hajeebhoy N, Martin L, Lapping K (2013). Using an evidence-based approach to design large-scale programs to improve infant and young child feeding. Food Nutr Bull.

[7] Nguyen PH, Menon P, Keithly SC, Kim SS, Hajeebhoy N, Tran LM (2014). Program impact pathway analysis of a social franchise model shows potential to improve infant and young child feeding practices in Vietnam. J Nutr.

[8] Whitington D (1998). Administering contigent valuation surveys in developing countries. World Dev.

[9] Diener A, O'Brien B, Gafni A (1998). Health care contingent valuation studies: a review and classification of the literature. Health Econ.

[10] FAO (2000). Applications of the contingent valuation method in developing countries.

[11] Dong H, Kouyate B, Cairns J, Sauerborn R (2003). A comparison of the reliability of the take-it-or-leave-it and the bidding game approaches to estimating willingness-to-pay in a rural population in West Africa. Soc Sci Med.

[12] Frew EJ, Wolstenholme JL, Whynes DK (2004). Comparing willingness-to-pay: bidding game format versus open-ended and payment scale formats. Health Pol.

[13] Menon P, Rawat R, Ruel M (2013). Bringing rigor to evaluations of large-scale programs to improve infant and young child feeding and nutrition: the evaluation designs for the Alive & Thrive initiative. Food Nutr Bull.

[14] Nguyen P, Tran L, Rawat R, Menon P (2014). Vietnam Endline Impact Evaluation Report.

[15] Mcintosh E, Mcintosh E, Clarke P, Frew EJ, Louviere JJ (2010). Applied methods of cost – benefit analysis in health care. Health Economic Evaluation.

[16] Olsen S (2001). Theory versus practice: a review of “willingness to pay in health and health care.”. Health Econ.

[17] Van Minh H (2013). Costs of providing nutrition examination services and integrated management of severe acute malnutrition in Vietnam.

[18] Phusit P (2006). An application of the asset index for measuring household living standards in Thailand. International Health Policy Program (IHPP), Bangkok, Thailand.

[19] Cawley J (2008). Contingent valuation analysis of willingness to pay to reduce childhood obesity. Econ Hum Biol.

[20] Engelhardt K, Ahn BC, Cho SI, Joung H (2007). Predictors of interest in nutrition topics and willingness to participate in local nutrition programmes. J Public Health.

[21] Segre J, Winnard K, Abrha TH, Abebe Y, Shilane D, Lapping K (2012). Willingness to pay for lipid-based nutrient supplements for young children in four urban sites of Ethiopia. Maternal Child Nutr.

[22] A&T (2014). Cost of providing nutrition counselling service in Vietnam.

[23] Nguyen PH, Kim SS, Keithly SC, Hajeebhoy N, Tran LM, Ruel MT (2014). Incorporating elements of social franchising in government health services improves the quality of infant and young child feeding counselling services at commune health centres in Vietnam. Health Policy Plann.

[24] Decree 85/2012/ND-CP: decree on operational, financial mechanisms and health service cost for Goverment health facilities (2012) http://vanban.chinhphu.vn/portal/page/portal/chinhphu/hethongvanban?class_id=1&mode=detail&document_id=164055.

[25] Whittington D (1998). Administering contingent valuation surveys in developing countries. World Dev.

[26] Onwujekwe O, Nwagbo D (2002). Investigating starting-point bias: a survey of willingness to pay for insecticide-treated nets. Soc Sci Med.

[27] Milanesi J (2010). Measuring demand for sanitation in developing countries: a new theoretical and methodological framework for contingent valuation surveys.

[28] O'Brien B, Viramontes JL (1994). Willingness to pay: a valid and reliable measure of health state preference?. Med Decis Making.

[29] Griffin C, Briscoe J, Singh B, Ramasubban R, Bhatia R (1995). Contingent valuation and actual behavior: predicting connections to new water systems in the State of Kerala, India. World Bank Econ Rev.

[30] Kahneman D (2012). Thinking, fast and slow.

